# 二维离子排斥-离子交换色谱配合阀切换技术测定G2级磷酸中的痕量阴离子

**DOI:** 10.3724/SP.J.1123.2024.04010

**Published:** 2025-03-08

**Authors:** Jiading WANG, Gao TANG, Yan LU, Hengjun LU, Tao YE

**Affiliations:** 1.中国计量大学材料与化学学院, 浙江 杭州 310018; 1. College of Materials and Chemistry, China Jiliang University, Hangzhou 310018, China; 2.浙江方圆检测集团股份有限公司, 浙江 杭州 310018; 2. Zhejiang Fangyuan Test Croup Co., Ltd., Hangzhou 310018, China

**Keywords:** 电子级磷酸, 痕量阴离子, 阀切换, 二维离子色谱, electronic grade phosphoric acid, trace anions, valve switching, two-dimensional ion chromatography (2D-IC)

## Abstract

本研究建立了在超净环境下基于二维离子排斥-离子交换色谱配合阀切换技术测定G2级磷酸中痕量阴离子含量的方法。通过比较确定了第一维超纯水流经离子排斥柱的最佳柱流速为0.5 mL/min,并确定阀切换窗口,以尽可能降低PO_4_^3-^在阴离子富集柱上的富集水平,减小了磷酸基体的干扰;采用抑制器外接水模式,降低了基线噪声;优化了第二维的淋洗程序,实现了痕量目标阴离子的良好分离,避免了硝酸根的假阳性风险。结果表明:Cl^-^、Br^-^、NO_3_^-^和SO_4_^2-^ 4种阴离子在0.5~20 μg/kg含量范围内呈现良好的线性关系,相关系数>0.999;检出限和定量限分别为0.09~0.29 μg/kg和0.29~0.97 μg/kg。在4个添加水平下,各阴离子的加标回收率为91.7%~103.6%,RSD为0.1%~4.9%。本方法具有重复进样稳定性好、检出限低的优势,方法的精密度和准确度高,能满足G2级磷酸中痕量阴离子的检测需求。此外,通过单一标准物质保留时间定性,本研究在G2级磷酸中发现除常规阴离子外,可能还存在以亚磷酸根(HPO_3_^-^)和六偏磷酸根(PO_3_^-^)_6_为代表的其他磷系阴离子和阴离子簇。这一发现对于今后更高纯度磷酸的纯化工艺开发具有一定的理论和应用意义。

## Abstract (en)

This study established a method for determining trace anions in G2-grade phosphoric acid under ultra-clean conditions that used two-dimensional ion-exclusion-ion-exchange chromatography coupled with valve-switching technology. The optimal flow rate of ultrapure water through the ion-exclusion column in the first dimension was determined to be 0.5 mL/min through comparative experiments. The valve-switching window was optimized to minimize enriching PO_4_^3-^ on the anion-enrichment column and reduce interference from the phosphoric-acid matrix. The use of external water mode for the suppressor led to lower baseline noise, and optimizing the elution program of the second dimension enabled target trace anions to be well separated, thereby avoiding the risk of a false positive for NO^3-^. The anions of Cl^-^, Br^-^, NO^3-^, and SO_4_^2-^ exhibited good linearities within the 0.5-20 μg/kg, with correlation coefficients ≥0.999. The limits of detection (LODs) and quantification (LOQs) were 0.09-0.29 μg/kg and 0.29-0.97 μg/kg, respectively. The spiked recoveries of each anion at four spiked levels ranged from 91.7% to 103.6%, with RSDs of 0.1%-4.9%. The developed method demonstrated excellent stability through repeated injections, and low detection limits; it is highly precise and accurate, and meets the detection requirements for trace anions in G2-grade phosphoric acid. Additionally, retention-time comparisons with single standard substances led to the discovery of other phosphorus-related anions and anion clusters, such as hypophosphite (HPO_3_^-^) and hexametaphosphate (PO_3_^-^)_6_, in G2-grade phosphoric acid, in addition to conventional anions. This finding is theoretically and practically significant for the future development of purification processes for higher-purity phosphoric acid.

电子级磷酸是半导体行业中的关键性超净高纯化学品之一,广泛用于半导体制程中的硅片和晶圆清洗、光刻过程中对Si_3_N_4_膜和铝膜的蚀刻等工艺中^[1,2]^,其需求量逐年增长,而国内集成电路和薄膜晶体管液晶显示器产业用磷酸基本依赖进口,且相应质量管控标准的建立和检测方法开发相对滞后^[3]^。磷酸试剂的品质直接影响芯片成品率,残留的阴离子若附着于晶圆表面可能导致漏电流等故障^[4]^。为提高制程的良品率,国际半导体设备与材料组织(SEMI)对电子级磷酸的等级标准做出统一规范,将电子级磷酸的纯度等级划分为G1~G3级,并对G1~G3级磷酸中Cl^-^、NO_3_^-^和SO_4_^2-^做出一致限量要求,分别为1、5和12 mg/kg^[5]^。随着半导体制程的发展,硅晶圆特征线宽不断缩小,对磷酸的纯度和杂质管控更为严格,因此对电子级磷酸中阴离子的检测方法也提出了更高灵敏度的要求。

目前磷酸中痕量阴离子的测定方法有比色法^[6]^、目视比浊法^[7]^和分光光度法^[8]^,这类手工离线测定方法往往前处理过程耗时费力且定量准确度不高,无法应用于痕量阴离子的测定。若用常规离子色谱方法直接进样测定,过量的磷酸基体极易造成色谱柱过载。因此常规离子色谱法测定弱酸时通常采用固相萃取^[9]^、电解^[10]^和大比例稀释进样^[11]^等来消除基体干扰,但这类离线前处理方式操作较为复杂,额外增加了杂质引入的风险。其中大比例稀释直接进样的方式仅能适用于普通等级的磷酸,若对G2级磷酸进行大比例稀释除直接影响目标离子的检测灵敏度外,背景离子或杂质的相对浓度可能增加,从而影响定量结果的准确度。为解决这类问题,二维离子排斥-离子交换色谱法(ICE-IC)在线预处理技术相对优势较大,在氢氟酸^[12]^、磷酸^[13⇓-15]^、有机酸^[16]^和其他弱酸中的痕量检测已有相关报道。但此前相关研究多在常规试验环境中进行,无法屏蔽实验用水及实验环境污染,且应用于实际G2级磷酸样品测试时无法实现基体中部分未知杂质离子与待测目标阴离子的完全分离,存在一定的假阳性风险,现有文献方法显然已无法满足高纯磷酸中痕量阴离子的检测需求。

本研究在超净环境下应用二维阀切换系统对G2级磷酸中的痕量阴离子进行定量分析。采用抑制器外接水模式实现更低基线噪声,采用阀切换技术实现大部分磷酸基体的在线消除,配合优化后的淋洗条件实现目标阴离子和其他磷系杂质阴离子的分离,达到优于μg/kg级目标阴离子的准确定量测定。本研究对其他弱电离酸中痕量阴离子的测定也具有一定的参考意义。

## 1 实验部分

### 1.1 实验环境

本研究的实验在动态千级洁净实验室内进行(ISO Class 5,恒温(20±1) ℃,恒湿45%~55%RH (相对湿度),空气中≥0.5 μm的微粒个数≤3520 个/m^3^),样品配制与保藏环境为动态百级环境^[17]^,内部环境杂质离子的潜在污染可以控制在极低水平。

### 1.2 仪器及试剂

二维离子色谱仪(Dionex Aquion RFIC,美国ThermoFisher公司),内置阀切换系统和KOH淋洗液发生器,辅助进样系统配AXP泵和离子捕获柱(Dionex IonPac ATC-HC 500, 250 mm×9 mm),第一维配离子排斥柱(Dionex IonPac ICE-AS1, 250 mm×9 mm)和阴离子富集柱(Dionex IonPac UTAC-XLP2, 16 mm×6 mm),第二维配Dionex IonPac AS11-HC保护柱(50 mm×4 mm)和分析柱(250 mm×4 mm)、抑制器(Dionex ADRS 600 Carbonate 4 mm),所有数据处理操作均在Chromeleon 7.0色谱工作站进行;十万分之一精密电子分析天平(Mettler Toledo MS105DU,美国梅特勒公司)。

市售1000 mg/L阴离子标准溶液(Cl^-^、Br^-^、NO_3_^-^、ClO_3_^-^ 、SO_4_^2-^,美国O2si公司); G2级磷酸(上海傲班科技有限公司);超纯水(Milli-Q IQ-7000, EW-1电子级超纯水系统,18.2 MΩ·cm,美国Millipore公司);高品质特氟龙(PFA)试剂瓶(上海亚速旺商贸有限公司);高纯氮气(法国液态空气集团)。

### 1.3 样品的制备

1%(质量分数)磷酸溶液:精密称取适量G2级磷酸样品至预先用超纯水浸泡过夜的PFA瓶中,用超纯水以称量法稀释100倍。33%(体积分数)磷酸溶液:准确量取10 mL G2级磷酸样品,置于已用超纯水浸泡过夜的PFA瓶中,并以超纯水定容至30 mL。上述溶液充分混匀,待冷却至室温后即可进样分析。

### 1.4 标准溶液的配制

在配制标准溶液前,所有与样品或样品稀释液直接接触的器皿、量具等均需预先经超纯水充分润洗浸泡(包括但不限于取用标准溶液和样品的PFA枪头、盛放标准溶液的PFA瓶等),通过本底检查后方可用于本实验。

分别将1000 mg/L 5种阴离子标准溶液用移液枪移取50 μL置于去皮后的干燥PFA瓶中,并于十万分之一天平上准确读取加样量,待所有阴离子标准溶液称样完毕后,用称量法加入适量电子级超纯水,配制上述阴离子含量约为1000 μg/kg的阴离子混合标准溶液。再用标准加入法向1%(质量分数)磷酸溶液中准确加入适量1000 μg/kg阴离子混合标准溶液液,配制系列标准溶液,含量约为0.50、1.00、2.00、5.00、10.00和20.00 μg/kg,上述称样量均精确至0.02 mg,该阴离子系列标准溶液需现配现用。

### 1.5 色谱条件

二维离子色谱系统阀切换系统结构简图见图1,系统搭建时应尽量缩短仪器各单元之间的连接管道以减少系统死体积。第一维系统配1000 μL定量环,样品通过装在六通阀1上#2位的注射器反抽倒吸进入定量环,废液存储于注射器中从而避免二次污染。ATC-HC离子捕获柱用于进一步去除第一维去离子水中可能存在的阴离子杂质。在ICE-AS1离子排斥柱上实现目标离子与磷酸基体在线分离,目标离子富集于阴离子富集柱上,第二维配AS11-HC分析柱系统实现目标阴离子分离。分析流路淋洗液为氢氧化钾,抑制器模式为外接水模式,抑制电流为149 mA,电导池温35 ℃,柱温30 ℃,第一维淋洗液流速为0.5 mL/min,第二维柱流速为1.0 mL/min。

**图1:**
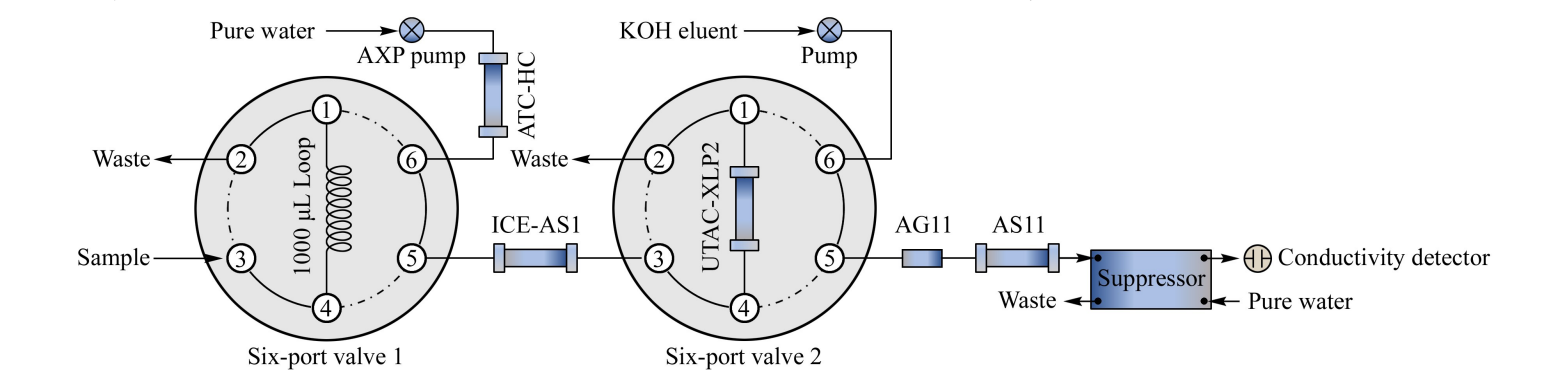
2D-IC阀切换系统结构示意图

### 1.6 阀切换系统工作程序

为便于系统阀切换时间的计量,工作程序以样品装载至定量环时刻作为起始时间-9 min(见表1)。工作程序开始前六通阀1处于Load状态,通过注射器在六通阀1上#2位手动吸取样品使之充分润洗定量环,以超纯水平衡ICE-AS1离子排斥柱并冲洗管路,六通阀2处于Inject状态,以KOH淋洗液平衡富集柱和分析流路色谱柱。工作程序开始时六通阀1位于Inject状态,此时AXP泵用超纯水将定量环中的样品推入ICE-AS1柱,由于强电离的目标阴离子受Donnan排斥率先于-2 min开始被洗脱,此时六通阀2切至Load状态实现对目标离子的富集。待目标离子富集完成后,为避免大量磷酸基体随后洗脱至富集柱被富集,在0 min时六通阀2切回Inject状态,KOH淋洗液将富集柱中富集的阴离子洗脱至第二维分析柱系统进行分析测定。

**表1:** 分析系统淋洗液梯度及阀切换工作程序表

Time/min	c(OH^-^)/(mmol/L)	Valve 1	Event of 1D system	Valve 2	Event of IC system
-9.0	8.0	inject	/	inject	analyzing system balance
-2.0	8.0	inject	matrix separation window start	load	target anion enrichment
0	8.0	inject	matrix separation window end	inject	target ion analysis
5.0	8.0	load	preparing a new sample for injection	inject	target ion analysis
10.0	8.0	load	equilibrium exclusion column	inject	target ion analysis
60.0	60.0	load	equilibrium exclusion column	inject	target ion analysis
63.0	60.0	load	equilibrium exclusion column	inject	target ion analysis
63.1	8.0	load	equilibrium exclusion column	inject	target ion analysis

## 2 结果与讨论

### 2.1 第一维色谱条件的确定

#### 2.1.1 确定第一维淋洗液的最佳流速

第一维淋洗液流速直接影响基体离子和目标离子在离子排斥柱上的分离效果,同时也决定了进入第二维的基体离子水平。因此应选择合适的第一维淋洗液流速以在目标阴离子的富集效率与减少进入富集柱的磷酸基体水平之间取得最佳平衡,从而优化整体分析效果。本实验将离子排斥柱与检测器直接相连,在0.3、0.4、0.5和1 mL/min流速下分别测试33%(体积分数)磷酸溶液和1000 μg/kg的阴离子混合溶液,所得色谱图见图2。

**图2:**
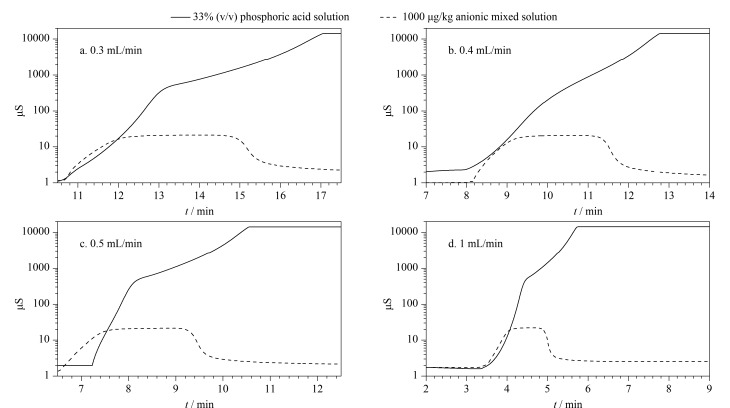
不同流速下阴离子混合溶液和磷酸溶液在离子排斥柱上的洗脱情况

结果表明在前述4种选定流速条件下,均无法完全分离磷酸基体和目标阴离子。不同流速下进入第二维富集柱的磷酸基体水平各有差异,因此通过比较不同流速下磷酸基体的峰面积大小、基线的状态以及待测离子在分析柱上的保留行为,从而确定最佳第一维淋洗流速。实验部分,将离子排斥柱接入第二维分析柱系统,根据图2确定不同流速下目标阴离子在排斥柱上的洗脱时间,0.3、0.4、0.5和1 mL/min流速下分别在10.5~16.5、8~12.5、7~10和3.5~5.5 min进行阀切换截取对应馏分在富集柱中富集,最后以KOH淋洗液引入分析柱系统测定。实验结果显示,在流速为0.3 mL/min时进入富集柱的磷酸基体过多,易造成基线抬升干扰目标阴离子测定(见图3a)。而在0.4 mL/min流速下,磷酸基体对目标阴离子的保留时间产生一定偏移(见图3b)。当流速为0.5 mL/min和1 mL/min时,目标阴离子和磷酸基体富集水平几乎一致,且不会对目标阴离子的保留行为造成干扰(见图3c和3d)。为降低第一维外加泵用去离子水的更换频率,最终确定以0.5 mL/min作为第一维淋洗流速。

**图3:**
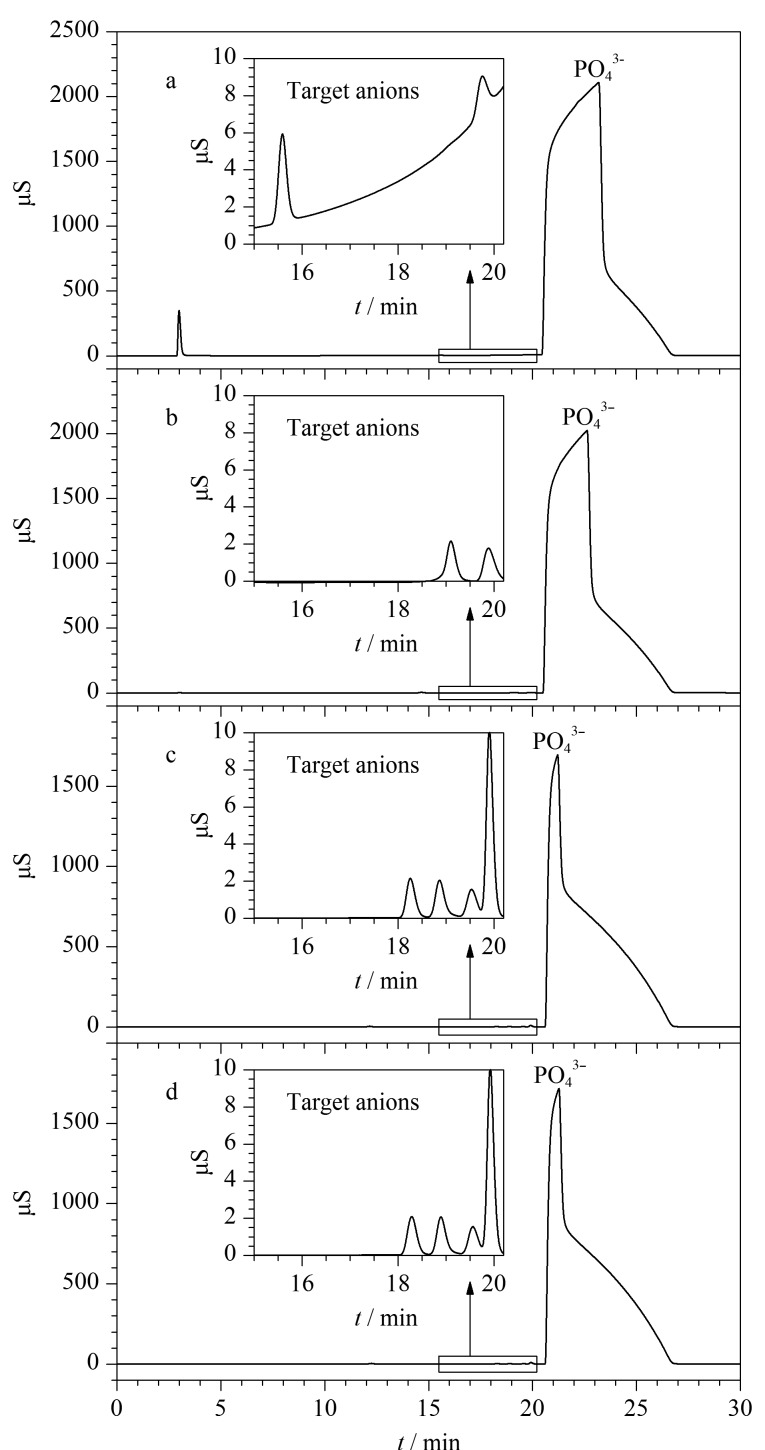
不同流速下阴离子磷酸混合溶液的第一维色谱图

#### 2.1.2 确定基体分离的最佳阀切换窗口

根据图2c实验设置六通阀1在7 min时切换至Inject状态,同时六通阀2切换至Load状态使目标阴离子在富集柱上富集,10 min时六通阀2切换至Inject状态,将富集柱上富集的阴离子冲洗至第二维分离系统进行分析。结果发现,该条件在首次进样33%(体积分数)磷酸溶液时分析效果较好,色谱图也未见异常(见图4a),但在连续进样测试第二针时会出现基线抬高、保留时间偏移等现象,进而会影响SO_4_^2-^定量(见图4b放大部分)。

**图4:**
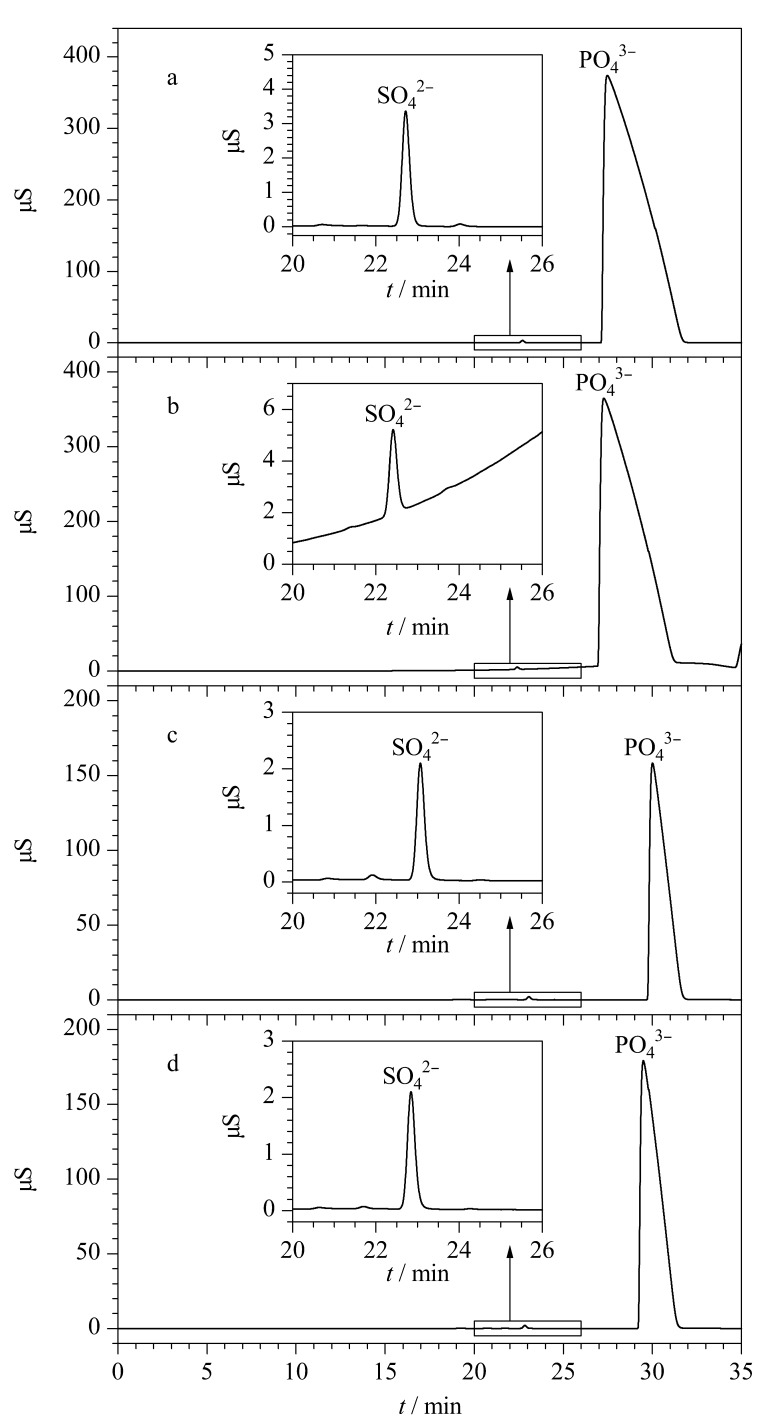
两种阀切换窗口下连续分析33%(体积分数)磷酸溶液的色谱图

上述结果表明,在切换窗口为7~10 min时阀切换窗口过宽,导致大量磷酸基体进入第二维柱系统。因此实验进一步比较了7~8.5、7~9和7~9.5 min 3种阀切换窗口对磷酸基体在线消除效果的影响。结果显示,在7~8.5 min窗口下,目标阴离子和磷酸基体大部分被切除至废液,导致响应信号偏低。在7~9.5 min窗口下,仍存在大量磷酸基体共同洗脱进入富集柱。经过分析,当切换窗口的时间为7~9 min时,能够在兼顾待测阴离子响应信号的同时,最大限度地避免磷酸基体富集,连续进样下基线状态平稳,不影响目标离子定量(见图4c和4d)。因此确定基体分离的最佳阀切换窗口为7~9 min,对应表1中-2~0 min。

### 2.2 抑制器抑制模式的选择

文献[13,15]中并未明确说明所使用的抑制器模式,因此无法确定哪种模式能提供更低的基线噪声。本实验比较了外接水模式和自动再生抑制器循环模式下的基线噪声差异。结果表明,在自动再生抑制器模式下测试33%(体积分数)磷酸溶液的基线噪声为0.0064 μS,而在外接水模式下测试33%(体积分数)磷酸溶液的基线噪声为0.0008 μS。分析发现,两种抑制器模式下基线噪声存在差异的原因为在自再生抑制模式下,磷酸基体通过抑制器的第二回路时易与其中的H^+^结合,导致抑制效率降低和检测器基线波动增加。因此,本实验选用外接水模式,以进一步降低基线噪声,从而实现更低的方法检出限。

### 2.3 初步确定第二维分析系统的淋洗条件

KOH最高淋洗浓度为35 mmol/L时,连续进样33%(体积分数)磷酸溶液,我们观察到SO_4_^2-^的保留时间发生了偏移,保留时间的相对标准偏差(RSD)为0.52%(*n*=5)。同时,基线也出现了一定程度的抬升,影响了离子定量的准确性,SO_4_^2-^峰面积RSD为4.7%(*n*=5)。为避免磷酸基体造成的保留时间偏移和基线抬升的干扰,实验通过提升淋洗液浓度来充分洗脱在分析流路中强保留的磷酸基体,淋洗梯度从开始的8 mmol/L(0 min)开始,经过10 min保持稳定,然后在接下来的25 min内线性增加至60 mmol/L(35 min),并提升抑制器电流至149 mA,以电解更多H^+^,匹配淋洗梯度的变化。在优化后的淋洗条件下连续进样33%(体积分数)磷酸样品,实验结果如下:SO_4_^2-^保留时间RSD为0.01%(*n*=5),峰面积RSD为2.73%(*n*=5),结果表明在连续进样过程中目标离子的保留时间基本恒定,且基线不会抬升,说明在此淋洗梯度下没有目标离子或磷酸基体在柱系统中积累,避免了系统积累效应,确保每次进样条件一致,结果重复性良好。

### 2.4 避免NO_3_^-^假阳性检出的实验条件确定

由于不同的生产工艺,磷酸中引入的阴离子种类各异。以2.3节确定的淋洗梯度,对1% (体积分数)的G2级磷酸溶液中加入约20 μg/kg阴离子混合标准溶液进行测试,发现存在未知离子干扰NO_3_^-^定量(见图5)。因此对于本实验的G2级磷酸,如果在纯水基体中建立标准曲线进行目标离子的定量分析时,当存在保留时间偏差且未通过添加NO_3_^-^单一标准物质进行验证,可能将该未知离子误判为NO_3_^-^,从而存在一定的NO_3_^-^假阳性检出风险。而在磷酸基体下建立标准曲线进行目标离子的定量分析时,虽然可以避免NO_3_^-^的假阳性检出风险,但由于未实现基线分离,仍会受到未知阴离子的干扰,影响NO_3_^-^的定量准确性。通过单一标准物质保留时间比对,初步认为该未知离子很可能是亚磷酸根(HPO_3_^-^),推测其来源可能与磷酸生产过程中白磷的不完全氧化有关^[18]^,此外,单一标准物质的保留时间比对还显示出六偏磷酸根((PO_3_^-^)_6_)的存在,但具体定性仍有待后续通过质谱数据进一步验证。

**图5:**
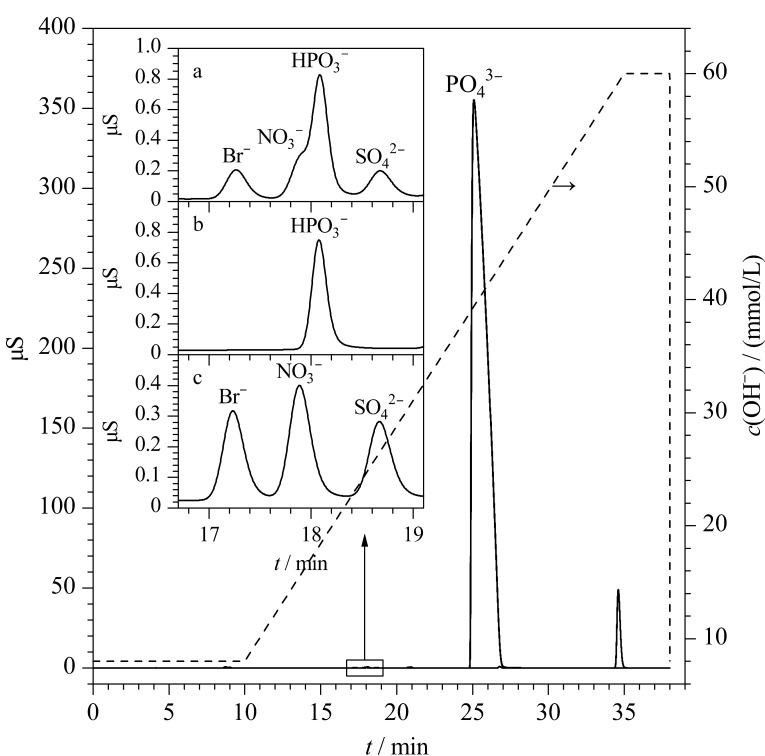
未知离子(可能是亚磷酸根)引起的NO_3_^-^假阳性干扰

AS11离子交换柱上离子的保留时间与离子价态、离子半径和离子极化呈正相关,由于在氢氧根系统下不同的淋洗强度会影响目标离子之间的分离度。为确保NO_3_^-^在磷酸基体中的定量准确度,对淋洗梯度进行如下优化:淋洗液浓度从0 min时的8 mmol/L开始,经过10 min保持稳定,然后在接下来的50 min内线性增加至60 mmol/L(60 min),这一优化使得淋洗液浓度更缓慢地上升,从而实现了1%(质量分数)G2级磷酸溶液中NO_3_^-^与上述未知离子的良好基线分离(见图6)。此外这一步骤还确保了磷酸基体在分析流路中的充分洗脱,使分析柱和保护柱在分析下一针样品前能恢复到分析平衡状态,进一步提高了方法的稳定性和重复性。

**图6:**
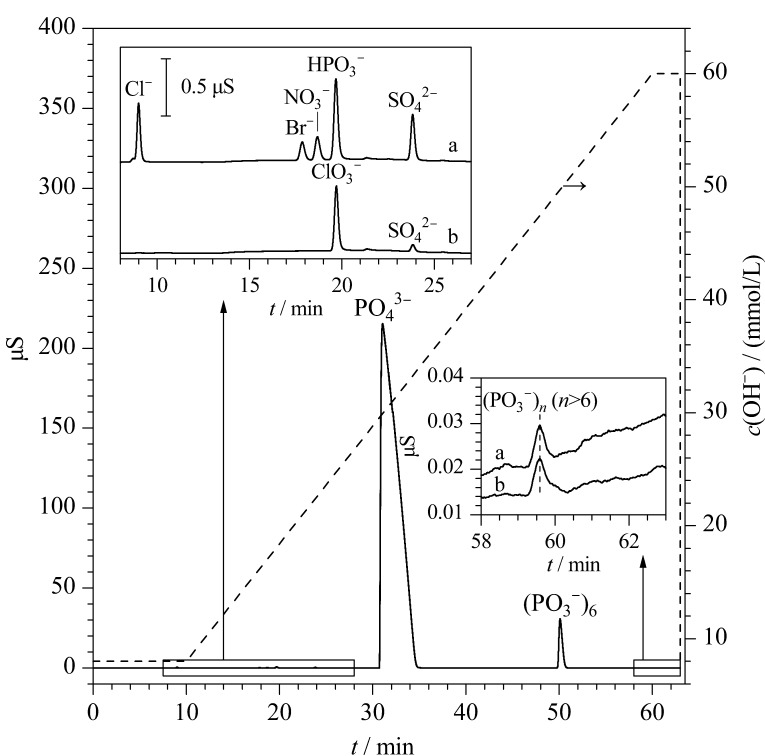
最终的淋洗梯度及样品加标测试图

### 2.5 方法学验证

#### 2.5.1 工作曲线、检出限和定量限

待1.4节通过标准加入法配制的系列标准溶液冷却至室温后,按照1.6节淋洗程序依次进样分析,测定各离子的色谱峰面积。以加入的离子含量为横坐标*x*,峰面积为纵坐标*y*,绘制标准曲线。结果表明,4种离子在0.5~20 μg/kg范围内均呈现良好的线性关系,相关系数(*r*)>0.999,分别按照3倍和10倍*S/N*计算检出限和定量限。检出限和定量限分别为0.09~0.29 μg/kg和0.29~0.97 μg/kg,其中检出限较文献^[5,14,15,19]^报道低(见表2)。

**表2:** 4种阴离子的线性方程、线性范围、相关系数、检出限和定量限

Anion	Linear equation	Linear range/ (μg/kg)	r	LODs/(μg/kg)		LOQ/(μg/kg)
				This study	Literature	
Cl^-^	y=0.005x-0.0003	0.5-20	0.99974	0.09	3^[5]^, 60^[14]^, 12^[15]^, 7^[19]^	0.29
Br^-^	y=0.0024x-0.0003	0.5-20	0.99978	0.29	/	0.97
N	y=0.0031x+0.0004	0.5-20	0.99978	0.15	4^[5]^, 40^[14]^, 19^[15]^, 3^[19]^	0.50
S	y=0.0043x+0.0148	0.5-20	0.99978	0.16	90^[5]^, 680^[14]^, 30^[15]^, 3^[19]^	0.54

*y*: peak area; *x*: content, μg/kg.

#### 2.5.2 加标回收率和精密度

大多数市售电子级磷酸仅对金属杂质有管控要求,而对阴离子杂质未作严格限量,部分目标阴离子含量较高,超过了本实验的线性范围。实验按1.3节方法制备1%(质量分数)G2级磷酸溶液,向其中加入适量阴离子混合标准溶液,配制成4组加标含量分别约为0.50、4.00、10.00和16.00 μg/kg的溶液。同一天内对上述4组溶液分别连续测定5次。根据测定结果计算,Cl^-^、Br^-^、NO_3_^-^和SO_4_^2-^的加标回收率为91.7%~103.6%,不同含量样品溶液的日内精密度结果均在5%以内(见表3),结果表明本方法加标回收率和精密度良好。

**表3:** 磷酸样品中阴离子在4个水平下的加标回收率(*n*=5)

Anion	Background/ (μg/kg)	Spiked/ (μg/kg)	Recovery/ %	RSD/ %
Cl^-^	0	0.52	91.7	4.9
	0	4.07	99.6	1.1
	0	10.18	100.0	0.4
	0	16.33	99.3	0.1
Br^-^	0	0.52	93.3	2.3
	0	4.13	93.2	2.0
	0	10.34	95.6	0.4
	0	16.59	99.3	0.2
N	0	0.52	94.0	2.8
	0	4.10	103.6	2.3
	0	10.28	101.2	0.4
	0	16.48	99.4	0.3
S	3.19	0.48	98.1	4.3
	3.12	3.76	96.2	1.3
	3.17	9.42	97.2	0.3
	3.17	15.11	98.9	0.2

## 3 结论

本研究借助千级洁净实验室内的超低背景配合离子排斥-离子交换二维阀切换系统成功实现了G2级磷酸基体中痕量阴离子的检测。在传统二维阀切换系统的基础上,本研究对第一维色谱柱流量、阀切换窗口进行了深入研究,优化后的切换窗口去除了绝大部分磷酸基体的干扰,在连续进样过程中能表现出较好的稳定性;优化了二维色谱淋洗浓度、淋洗时间和抑制器工作模式,确保离子间的分离度和极低的系统基线噪声水平。本方法在实际G2级磷酸样品测定中排除了目标离子(主要是硝酸根)的假阳性风险,且具有检出限低、精密度高的特点,有望在今后高纯电子级磷酸的方法标准化过程中作为首选方法,助力国产电子级磷酸的优化升级,实现国产化替代。同时本研究可为其他电子级弱电离酸或类似基体中痕量杂质离子的测定方法开发提供一定的参考。但本研究还存在部分不足之处:由于生产工艺的不确定性,实验中偶然发现的未知离子,目前仅通过单标在色谱中的保留时间进行了相对定性,未能采用质谱数据进行进一步的确证分析,因此无法充分确证未知离子,后续将通过质谱数据进行详细比对和验证。
